# A longitudinal study of *Angiostrongylus cantonensis* in an urban population of *Rattus norvegicus* in Brazil: the influences of seasonality and host features on the pattern of infection

**DOI:** 10.1186/1756-3305-7-100

**Published:** 2014-03-10

**Authors:** Raquel O Simões, Arnaldo Maldonado Júnior, Natalie Olifiers, Juberlan S Garcia, Ana Valéria FA Bertolino, José L Luque

**Affiliations:** 1 Curso de Pós-Graduação em Ciências Veterinárias, Universidade Federal Rural do Rio de Janeiro, Seropédica, RJ, Brazil; 2 Laboratório de Biologia e Parasitologia de Mamíferos Silvestres Reservatórios, Instituto Oswaldo Cruz, Av. Brasil 4365 Manguinhos, 21040-360 Rio de Janeiro, RJ, Brazil; 3 Departamento de Geografia, Universidade Estadual do Rio de Janeiro/Faculdade de Formação de Professores, Rua Dr. Francisco Portela, 1470, 24435-005 São Gonçalo, RJ, Brazil; 4 Departamento de Parasitologia Animal, Universidade Federal Rural do Rio de Janeiro, Caixa Postal 74540, 23851-970 Seropédica, RJ, Brazil

**Keywords:** *Rattus norvegicus*, *Angiostrongylus cantonensis*, pattern of infection, Brazil

## Abstract

**Background:**

The nematode *Angiostrongylus cantonensis* is a zoonotic parasite and the most important cause of eosinophilic meningitis worldwide in humans. In Brazil, this disease has been reported in the states of Espírito Santo and Pernambuco. The parasite has been detected in the naturally infected intermediate host, in the states of Rio de Janeiro, Pernambuco and Santa Catarina. The murid *Rattus norvegicus R. rattus* were recently reported to be naturally infected in Brazil. In this study, we conducted a two-year investigation of the dissemination pattern of *A. cantonensis* in *R. norvegicus* in an urban area of Rio de Janeiro state, Brazil, and examined the influence of seasonality, year, host weight and host gender on parasitological parameters of *A. cantonensis* in rats.

**Methods:**

The study was conducted in an area of Trindade, São Gonçalo municipality, Rio de Janeiro, Brazil. Prevalence of infected rats, intensity and abundance of *A. cantonensis* were calculated, and generalized linear models were created and compared to verify the contribution of host gender, host weight, year and seasonality to the variations in *A. cantonensis* abundance and prevalence in rats.

**Results:**

The prevalence of *A. cantonensis* infection was stable during the rainy (71%, CI 58.9- 81.6) and dry seasons (71%, CI 57.9-80.8) and was higher in older rats and in females. Seasonality, host weight (used as a proxy of animal age) and gender were all contributing factors to variation in parasite abundance, with females and heavier (older) animals showing larger abundance of parasites, and extreme values of parasite abundance being more frequent in the dry season.

**Conclusions:**

The high prevalence of this parasite throughout the study suggests that its transmission is stable and that conditions are adequate for the spread of the parasite to previously unaffected areas. Dispersion of the parasite to new areas may be mediated by males that tend to have larger dispersal ability, while females may be more important for maintaining the parasite on a local scale due to their higher prevalence and abundance of infection. A multidisciplinary approach considering the ecological distribution of the rats and intermediate hosts, as well as environmental features is required to further understand the dynamics of angiostrongyliasis.

## Background

The rat lungworm *Angiostrongylus cantonensis* is a nematode that, in its adult stage, parasitizes the pulmonary arteries of the synanthropic rodent *Rattus norvegicus*, the definitive host [1]. Mollusc species act as intermediate hosts and are infected by the first larval stage (L_1_) eliminated in rodent feces. Three weeks after mollusc infection, the larvae molt to the infective third stage (L_3_), becoming adult worms after being ingested by rats [2]. Humans become infected mainly by ingesting infected intermediate host or parts of the intermediate host consumed inadvertently when contaminating food is ingested, but may also be infected by consuming paratenic hosts (shrimps, crabs, frogs, planarians, and lizards) [3].

The recent introduction of *A. cantonensis* to the Americas [4] has resulted in human cases of eosinophilic meningitis throughout the continents [5-10]. In Brazil, this disease has been reported in the states of Espirito Santo, Pernambuco and São Paulo [7,11,12], and the natural intermediate host *A. fulica* has been observed in the states of Rio de Janeiro, Pernambuco and Santa Catarina [13,14]. Recently, *R. norvegicus* and *R. rattus* have been reported to be naturally infected in Brazil [15,16].

Although *A. cantonensis* is currently spreading rapidly throughout the Americas [4], there have not been any studies that have focused on the role of *R. norvegicus* in parasite transmission; instead, most studies have focused only on the intermediate host [17,18]. Horizontal studies are important because they allow us to characterize the profile of parasite transmission. In this study, we conducted a two-year investigation of *A. cantonensis* dissemination pattern in *R. norvegicus* in an urban area of Rio de Janeiro state, Brazil, and examined the influence of seasonality, year, host weight (used as a proxy for host age), and host gender on the prevalence and abundance of *A. cantonensis* in rats.

## Methods

### Study area

**Figure 1:**
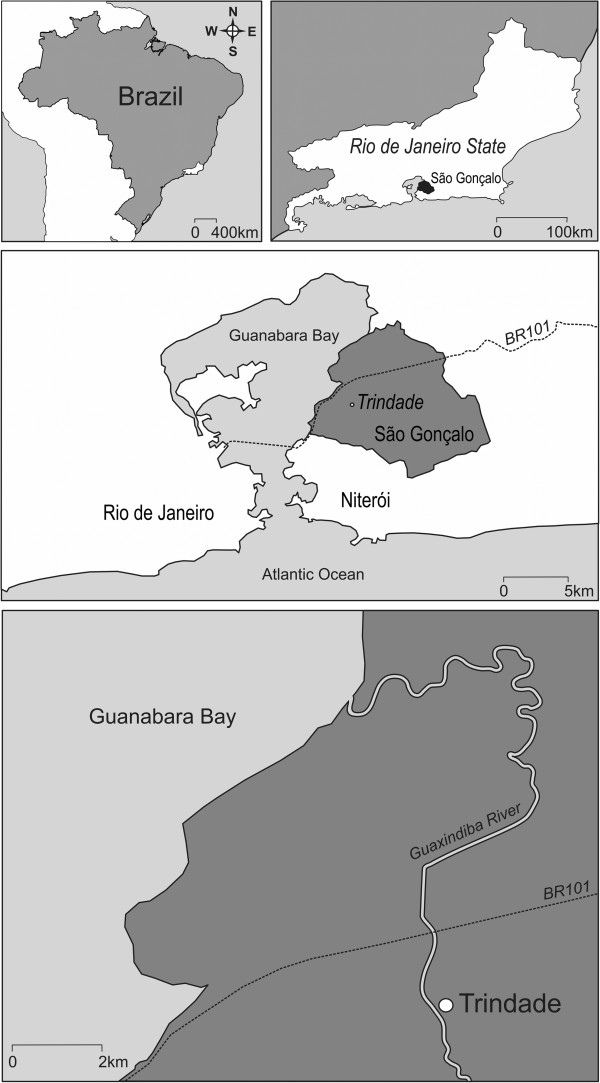
Map showing the study area.

The study was conducted in an urban area of Trindade, São Gonçalo municipality (22°48’26.7”S, 43°00’49.1”W), the second most populous city (~1 million habitants) in the state of Rio de Janeiro, Brazil (Figure 1). The climate is tropical with recognizable seasons: a rainy season from October to May and a dry season from April to November. The annual average temperature of the region is 25°C, with maximum and minimum temperatures ranging from 38°C to 17°C, respectively. The annual rainfall is 1200 mm (data obtained from Urban Climatological Station from Geosciences lab-LABGEO).

### Rodent capture

We established three transects spaced approximately 50 meters apart with 20 trapping stations each along polluted watercourse banks close to human habitats. A trapping station was established every 5 meters and included both a Tomahawk® trap (16 × 5 × 5 inches) and Sherman® trap (3 × 3.75 × 12 inches). The study was conducted every three months from March 2010 to December 2011 and each capture session lasted 4 consecutive days.

Captured rodents were transported to a field laboratory, where they were euthanized in a CO_2_ chamber, sexed, weighed and necropsied. All animal procedures followed the guidelines for capture, handling and care of mammals of the American Society of Mammalogists [19]. The collection permits for rodents were obtained from the Oswaldo Cruz Foundation (FIOCRUZ) Ethical Committee on Animal Use (Permit Number: LW 24/10) and the Brazilian government’s Institute for Wildlife and Natural Resources (Permit Number: 24353–1). Biosafety techniques were used during all procedures involving biological samples [20].

### Parasitological procedures

Helminths were collected from the pulmonary arteries and subarachnoid spaces. The organs were separated in Petri dishes and dissected under a stereomicroscope to remove the parasites. The collected worms were washed twice in physiological (0.9%) saline to remove tissue debris and were stored in 70% ethanol. Nematodes were clarified in lactophenol (40% lactophenol, 20% lactic acid, 20% phenol, and water q.s.p. 100 mL) and identified using a Zeiss Standard 20 light microscope. The morphology of the caudal bursa and size of the spicules were used as taxonomic characteristics for species identification according to Maldonado et al. (2010) [13] and Chen (1935) [21].

### Data analyses

Prevalences with their Sterne’s exact 95% confidence intervals (CI) [22] and the *k* index of aggregation were calculated using the program Quantitative Parasitology 3.0 [22]. For that, animals were divided into three age classes according to their weight: juveniles (<100 g), subadults (100–200 g) and adults (>200 g) [23]. A binary logistic regression was used to investigate the influence of season, year, host gender and host weight (used as a proxy of animal age) on the presence/absence of *A. cantonensis* in rats and a generalized linear model with a negative binomial distribution and log link was performed to verify the contribution of those variables to the observed variation in *A. cantonensis* abundance in pulmonary arteries of rats. We created models consisting of all combinations and interactions of predictors, as well as additional models containing an interaction between gender and weight plus year and/or season as additional variables. These additional models were included because *a priori* analyses pointed out the inclusion of the interaction “host gender* host weight” on the pool of best-fitting models (see below). All analyses were performed using PASW Statistics Version 18.0. Models were compared using the Akaike Information Criterion corrected for small sample size (AICc). Models were ranked based on the difference between the best approximating model (model with the lowest AICc) and all others in the set of candidate models (ΔAICc); models with differences within two units of the top model were considered competitive (best-fitting) models with strong empirical support [24]. The relative importance of each predictor (or interaction of predictors) was quantified by calculating relative variable weights (var. weight), which consist on summing the Akaike weights across all the models where the predictor occurs. To better investigate model fit, we calculated the likelihood ratio chi-square test for the best-fitting model of parasite abundance; we also calculated the Hosmer-Lemeshow statistic and computed the Nagelkerke R^2^ for the top model of parasite presence/absence. The wald Chi-Square test was used to check parameter significance in the best-fitting model.

We used the Mann–Whitney *U*-test and Moses test of extreme reaction in *post-hoc* analyses to test for differences in the parasite abundance and its range between host gender and seasons, whenever these predictors appeared amongst best-fitting models for parasite abundance. Spearman rank correlation was also used in *post-hoc* analyses to verify how parasite abundance varied with host weight in males and females. For all significance tests, α = 0.05.

## Results

One hundred and fourteen *R. norvegicus* were captured during the study. Fifty-six rats were collected during the rainy season (4 rats were not weighed) and 58 during the dry season (Table 1).

**Table 1:** **Number of *Rattus norvegicus *** **infected by *Angiostrongylus cantonensis *** **organized by age, sex and season**

Age*	**Rainy Season**		**Dry Season**		**Total**
	**Male**	**Female**	**Male**	**Female**	
Juvenile	2 (7)	2 (3)	2 (4)	0	6 (14)
Subadults	1 (1)	7 (8)	2 (6)	6 (8)	16 (23)
Adults	12 (17)	13 (16)	18 (25)	13 (15)	56 (73)

Positive rats (total number of collected rats).

*n = 110.

A total of 861 adult worms were recovered from pulmonary arteries and 82 young worms (L5) in the subarachnoid space found in all age class. The highest parasite burden (42 adult helminths) occurred in an adult female during the dry season. In addition, the distribution of the nematodes was aggregated (k = 2.04).

**Figure 2:**
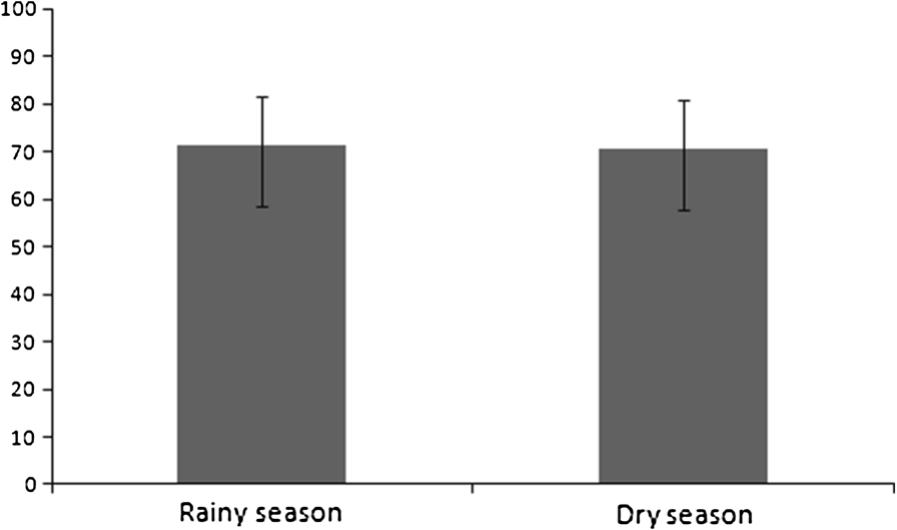
**Prevalence followed by 95% confidence interval (CI) of *Angiostrongylus cantonensis *** **in *Rattus norvegicus *** **collected during rainy and dry season in an urban area from Rio de Janeiro, Brazil.**

**Figure 3:**
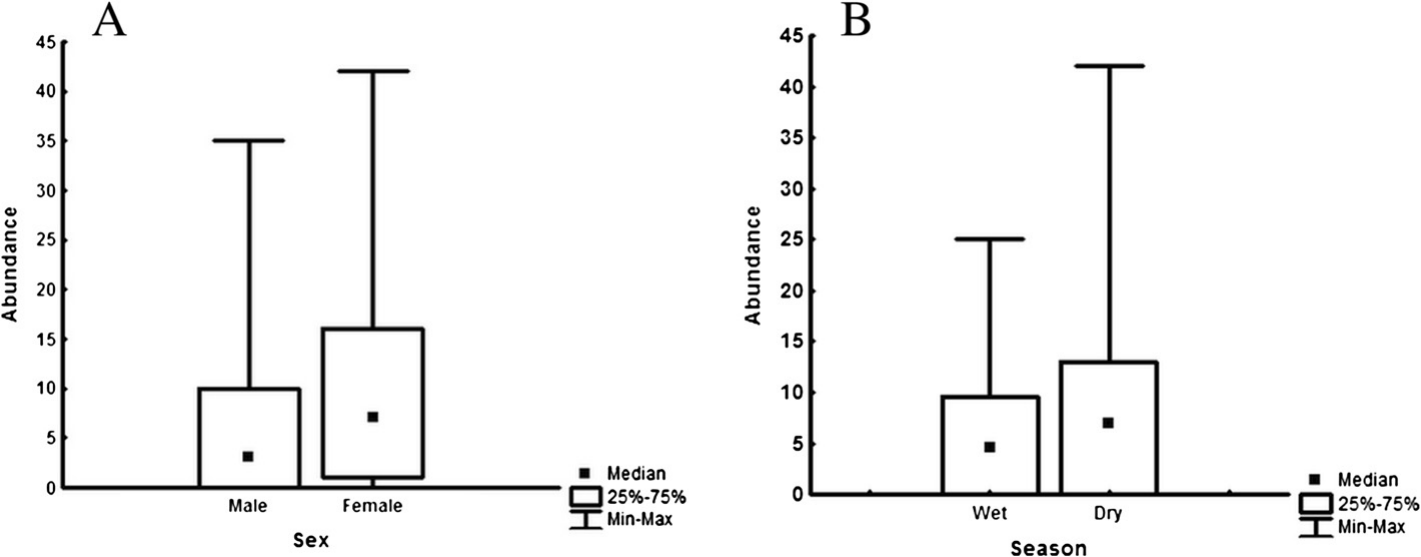
**Median, quartiles, and minimum and maximum values of abundance of *Angiostrongylus cantonensis *in *Rattus norvegicus *collected in an urban area from Rio de Janeiro, Brazil.** Values are given for **(A)** host gender and **(B)** season of data collection.

Four models were considered competing models in the analysis of parasite presence/absence in *R. norvergicus*, but their likelihoods were relatively low (from 0.12 to 0.19; Table 2). Moreover, model fit for the best-fitting model (model 1; Table 2) was also low (Nagelkerke R^2^ = 0.221; Hosmer-Lemeshow statistic: Chi-squared test = 8.561; df = 8; P = 0.381). Parasite presence/absence was best predicted by host weight (β_model 1_ = 0.006; var. weight = 0.54), host gender (β_female, model 1_ = 1.191; var. weight = 0.50), and year (β_2010, model 1_ = −0.693; var. weight = 0.50), although “year” was not a significant variable in the top model (Wald Chi-square = 2.20; df = 1; p = 0.138); All the other predictors in the best-fitting model were significant (P < 0.05). The prevalence of *A. cantonensis* varied from 63% to 87% (Table 3; Figure 2), and tended to be higher in females (78%; CI 65.2-87.2) than in males (59%; CI 46.4-70.1), while the prevalence in juveniles (43% CI 21.3-67.5) tended to be lower than in sub-adults (70% CI 48.9-84.5) and adults (77% CI 65.7-85.0). Prevalence was marginally larger in 2011, but confidence intervals overlapped considerably in this case (79% CI 66.4-88.1 against 64% CI 51.4-74.9; see also Table 3). The interaction between host weight and gender appeared in the third and fourth the best-fitting models for parasite presence/absence (Table 2), but its relative weight was lowest amongst predictors present in the best-fitting models (var. weight =0.40). Prevalence increases with age in both host sexes, but the difference between males and females occurs mostly in juveniles and subadults, with females showing larger prevalences than males. The multivariate analyses using host gender, host weight, year and season as independent variables and *A. cantonensis* abundance as the dependent variable indicated that two models were considered competitive (models 1 and 2; Table 4). Season, host weight and host gender contributed to variation in parasite abundance (likelihood ratio chi-square for model 1 = 16.03; df = 3; p = 0.001), even though Akaike weights indicated that the likelihood of the best-fitting models were relatively low (AICc weights = 0.40 and 0.15; Table 4). The variable “season” (var. weight = 0.87) appeared amongst the best-fitting models because 90% of the animals with abundance greater or equal than 20 worms (N = 11) were found in the dry season (β_dry season,,model 1_ = 0.579). Indeed extreme values of parasite abundance were more likely to occur in the dry season (Moses test of extreme reaction: P < 0.001; Figure 3B); parasite abundance, however, did not differ between seasons (P = 0.221). The interaction between host gender and host weight (var. weight = 0.57) appeared amongst the best-fitting models because the relationship between parasite abundance and host weight is stronger in females (β_females*weight, model 1_ = 0.004) than in males (β_males*weight, model 1_ = 0.002), and the β value for males was not significant (Wald Chi-Square = 2.971; df = 1; p = 0.085). All the other parameters in the top model were significant (P < 0.05). *Post-hoc* correlations between parasite abundance and host weight showed that *A. cantonensis* abundance increases with host weight in both sexes (R_females_ = 0.40, N = 50, P = 0.004; R_males_ = 0.30, N = 60, P = 0.002), which would explain why “host weight” (var. weight = 0.37) appeared as a main effect in the second best model (Model 2, Table 4).

**Table 2:** **Ranking of best-fit models describing parasite presence/absence in *Rattus norvegicus *** **captured at São Gonçalo, Rio de Janeiro/Brazil from 2010 to 2011; k = number of parameters in the models**

**Model**	**Log(l)**	**AICc**	**k**	**∆AICc**	**AICc weight**
1-Year + host gender + host weight	−54.30	116.98	4	0.00	0.19
2-Host gender + host weight	−55.41	117.05	3	0.08	0.18
3-Year + host gender × host weight	−54.35	117.08	4	0.10	0.18
4-Host gender × host weight	−55.80	117.82	3	0.85	0.12

**Table 3:** **Prevalence (95% confidence interval), median intensity, and mean abundance followed by the standard deviation of *Angiostrongylus cantonensis *** **in *Rattus norvegicus *** **collected during 2010 and 2011**

**Season**	**Month/year**	**Number of host**	**Prevalence**	**Median intensity**	**Mean abundance**
Rainy	March/2010	15	67% (41.5-85.0)	8 ± 5.7	6.2 ± 6.6
Dry	June/2010	15	67% (41.5-85.0)	18 ± 12.6	12.7 ± 13.5
	September/2010	19	63% (40.9-80.9)	11 ± 9.8	8.8 ±9.3
Rainy	December/2010	12	67% (51.7-93.2)	8.5 ± 5.1	6.4 ± 5.9
	March/2011	14	79% (38.6-83.8)	10 ± 7.7	6.6 ± 7.8
Dry	June/2011	15	87% (60.9-97.5)	10 ± 9.9	8.2 ± 9.9
	September/2011	10	70% (39.2-89.7)	8 ± 4.4	5.2 ± 5.1
Rainy	December/2011	14	79% (51.7-93.2)	7.5 ± 5	5.2 ± 5.5

**Table 4:** **Ranking of best-fit models describing parasite abundance in *Rattus norvegicus *** **captured at São Gonçalo, Rio de Janeiro/Brazil from 2010 to 2011; k = number of parameters in the models**

**Model**	**Log(l)**	**AICc**	**k**	**∆AICc**	**AICc weight**
1-Season + weight × gender	−331.53	671.4	4	0.00	0.40
2-Season + weight + gender	−332.47	673.3	4	1.89	0.15
3-Season + gender × weight + year	−331.51	673.6	5	2.16	0.13
4-Season + gender + weight + year	−332.35	675.3	5	3.83	0.06

Models 3 and 4 are presented for comparison purposes only.

Moreover, females had larger parasite abundance (P = 0.044) and extreme values of parasite abundance than males (P = 0.004; Figure 3A). Despite differences in parasite abundance between males and females, “host gender” as a main factor had the lowest relative variable weight amongst predictors in the best-fitting models (0.28).

## Discussion

The prevalence of *A. cantonensis* in *R. norvegicus* in Rio de Janeiro is relatively high compared to other localities where infected rats have been found [3]. As reviewed by Wang et al. (2008) [3], areas in Cuba and the Dominican Republic (both in the Americas) had a high prevalence of *A. cantonensis* at 60% (12 infected rodents of 20 collected) and 100% (5 infected rodents in 5 collected), respectively. However, the small sample sizes used in these short-term studies do not allow conclusive results and preclude inference from the helminth infrapopulation structure. In our study, we observed a high and stable prevalence of *A. cantonensis* over two years, which suggests that transmission is continuously high. Prevalences were marginally larger in 2011, but confidence limits overlapped considerably between years (see also Table 3). It would be interesting, however, to investigate whether *A. cantonensis* prevalence in *R. norvegicus* is gradually increasing in the long-term.

The recent settlement of *A. cantonensis* in the study area of São Gonçalo, Rio de Janeiro state could partially explain the high prevalence of this parasite. Additionally, the presence of the exotic intermediate host *A. fulica* in the study area [14], and its recent dispersion to all the 27 federations in Brazil [14,25,26] may contribute to the establishment and increase of *A. cantonensis* transmission to its vertebrate host. However, nothing is known about the population dynamics of the mollusc species present in the study area, although it has been demonstrated that variation in the structure of the intermediate host population plays an important role in the seasonal fluctuations in helminth community parameters in the definitive host [27]. Stability in prevalence may also be caused by the following: (1) a long parasite lifespan in the definitive host and liberation of L_1_ larvae over long periods of time, which may maintain parasite transmission to the intermediate host even in times of low snail abundance in the environment; (2) the presence of more than one mollusc species that is able to facilitate development to the infective larval stage L_3_[28]; (3) the lack of factors constraining rat abundance (e.g., constant and high food availability and refuge); and (4) *A. cantonensis* genetic heterogeneity [29,30], which may facilitate adaptation to new environments.

The positive relationship between *A. cantonensis* abundance and rat weight is at least partially associated with higher parasite burdens in older (and heavier) rats. As previously reported [31,32], this observation is likely due to the longer period of exposure to infection, which is also corroborated by the higher parasite prevalence found in adult rats. Additionally, the relatively weak relationship may also suggest the presence of a regulatory process for parasite density. Indeed, experimental studies demonstrated that rats exposed to *A. cantonensis* are able to modulate the parasite burden when re-infected [33].

Males have higher levels of testosterone and larger home ranges than females [34,35], which would potentially increase the probability of acquiring/maintaining an infection [36,37]. However, females had a higher prevalence and abundance of *A. cantonensis* than males, and the relationship between parasite abundance and host weight was also stronger in females, which somewhat undermines the hypothesis that males are more frequently exposed to infection because of their larger home ranges and/or testosterone levels. Although male mammals generally harbor more helminth parasites than females [37], the hormonal response of each gender may determine their distinct parasitic profiles [38]. Some studies have demonstrated female-biased parasitism [39-41], suggesting that sex-biased parasitism is a complex phenomenon influenced by hormones other than testosterone [39] and that additional variables in the host-parasite relationship can influence predisposition to infection and parasite burdens. For example, female adolescents seem to locomote more and spend more time exploring aversive areas than males of the same age [42]; if that implies that females explore their home ranges better, then they may be more prone to infection than males. This would explain why prevalences are particularly higher in juvenile and subadult females than males (see Table 3). Notwithstanding, our findings indicate that females may be particularly important for maintaining the parasite at a local scale due to their higher prevalence and abundance of infection and their philopatric behavior, whereas dispersion of the parasite to new areas may be mediated mainly by males that tend to have larger dispersion ability [43,44].

Although the prevalence of *A. cantonensis* did not vary between seasons (Table 3), the range of parasite abundance was greater during the dry season. Because the mollusc intermediate hosts are susceptible to desiccation [45], we would expect a lower abundance of snails and a consequently lower prevalence and abundance of *A. cantonensis* in rats during that season. The presence of a larger number of rats with young larvae of *A. cantonensis* in the subarachnoid space during the rainy season suggests that infection rates may actually be higher during the wet season. In many natural populations, however, animals show seasonal changes in stress hormones as a response to environmental changes (e.g. food or water shortage) and/or biotic factors (e.g. increased intraspecific competition) [46]. If stress hormones in rats were high during the dry season, then individuals may be less able to modulate parasite infection, which would explain the rats with higher burdens in that season. Nevertheless, there are no studies of seasonal changes in glucocorticoids in tropical mammals to support this hypothesis.

In summary, our results demonstrated that mainly host gender and host age (measured as host weight) contributed to variation in *A. cantonensis* prevalence, while season, host gender and host weight influenced parasite abundance. However, model fit for both parasite abundance and prevalence was low, which means that additional variables not investigated may be relatively more important for determining parasite abundance/prevalence in the definitive host. For instance, variables directly linked to infection rates and the abundance of the intermediate hosts might better predict *R. norvegicus* infection rates. Likewise, the host immune system and concomitant infection with other parasites may also be important predictors for the abundance of *A. cantonensis* in *R. norvegicus*. To disentangle the influence of these variables on parasite abundance and prevalence, field studies need to be associated with experimental studies.

## Conclusions

The stability of *A. cantonensis* prevalence throughout the duration of this study confirms that this parasite is established in the urban region of São Gonçalo municipality in Rio de Janeiro State and is under adequate conditions for spreading to other areas. We suggest that a multidisciplinary approach that considers ecological aspects (e.g.*,* variation in diet, dispersal ability and home range) of the rats and intermediate hosts, as well as environmental features is required to further understand the dynamic of several zoonoses, including angiostrongyliasis [47]. These studies are therefore essential for the implementation of surveillance and control strategies to reduce the risk of angiostrongyliasis among local residents and to limit the occurrence of new foci.

## Competing interests

The authors declare that they have no competing interests.

## Authors’ contributions

Conceived and designed the work: ROS, AMJ and JLL. Performed the work: ROS and JSG. Analyzed the data: ROS, NO, AMJ and JLL. Revised the manuscript for important intellectual content: AMJ, NO, JSG and JLL. Wrote the paper: ROS. All authors read and approved the final version of the manuscript.

## References

[B1] Acha PN Szyfres B Zoonoses and Communicable Diseases Man and Animals. Volume 3 2003 3 Washington, DC: Pan American Health Organization, Scientific and Tech. Publications

[B2] Wang QP Wu ZD Wei J Owen RL Lun ZR Human *Angiostrongylus cantonensis*: an update Eur J Clin Microbiol Infect Dis 2012 35 389 395 21725905 10.1007/s10096-011-1328-5

[B3] Wang Q Lai D Zhu X Chen X Lun Z Human angiostrongyliasis Lancet Infect Dis 2008 8 621 630 doi:10.1016/S1473-3099(08)70229-9 10.1016/S1473-3099(08)70229-9 18922484

[B4] Maldonado AJ Simões R Thiengo S Morales-Lorenzo J Angiostrongyliasis in the Americas Zoonosis. Volume 1 2012 1 InTech 303 320 Available from: http://www.intechopen.com/books/zoonosis/angiostrongyliasis-in-the-americas

[B5] Pascual J Bouli R Aguiar H Eosinophilic meningoencephalitis in Cuba, caused by *Angiostrongylus cantonensis* Amer J Trop Med Hyg 1981 30 960 962 7283014 10.4269/ajtmh.1981.30.960

[B6] Dorta-Contreras A Padilla-Docal B Moreira J Robles L Aroca J Fernando Alarcón F Bu-Coifiu- Fanego R Neuroimmunological findings of *Angiostrongylus cantonensis* meningitis in ecuadorian patients Arq Neuro-Psiquiatr 2011 69 466 469 10.1590/S0004-282X2011000400011 21755123

[B7] Lima A Mesquita S Santos S Aquino E Rosa L Duarte F Teixeira A Costa Z Ferreira M Alicata disease: neuroinfestation by *Angiostrongylus cantonensis* in Recife, Pernambuco, Brazil Arq Neuro-Psiquiatr 2009 67 1093 1096 10.1590/S0004-282X2009000600025 20069226

[B8] Barrow K St Rose A Lindo J Eosinophilic meningitis: is *Angiostrongylus cantonensis* endmic in Jamaica? West Indian Med J 1996 45 70 71 8772400

[B9] New D Little M Cross J *Angiostrongylus cantonensis* infection from eating raw snails New Eng J Med 1995 332 1105 1106 7898546 10.1056/NEJM199504203321619

[B10] Aguiar P Morera P Pascual J First record of *Angiostrongylus cantonensis* in Cuba Amer J Trop Med Hyg 1981 30 963 965 7283015 10.4269/ajtmh.1981.30.963

[B11] Espirito-santo MC Pinto PLS Mota DJG Gryschik RCB The first case of *Angiostrongylus cantonensis* eosinophilic meningitis diagonesd in the city of São Paulo, Brazil Rev Inst Med Trop 2013 55 129 132 doi:10.1590/S0036-46652013000200012 10.1590/S0036-46652013000200012 23563769

[B12] Caldeira R Mendonça C Gouveia C Lenzi H Graeff-Teixeira C Lima WS Mota EM Pecora IL Medeiros AMZ Carvalho OS First record of molluscs naturally infected with *Angiostrongylus cantonensis* (chen, 1935) (nematoda: metastrongyloidea) in Brazil Mem Inst Oswaldo Cruz 2007 102 887 889 10.1590/S0074-02762007000700018 18094889

[B13] Thiengo S Maldonado A Mota E Torres E Caldeira R Carvalho OS Oliveira AP Simões RO Fernandez MA Lanfredi RM The giant African snail *Achatina fulica* as natural intermediate host of *Angiostrongylus cantonensis* in Pernambuco, northeast Brazil Acta Trop 2010 115 194 199 10.1016/j.actatropica.2010.01.005 20083081

[B14] Maldonado A Jr Simões R Oliveira A Motta E Fernandez M Pereira ZM Monteiro SS Torres EJ Thiengo SC First report of *Angiostrongylus cantonensis* (Nematoda: Metastrongylidae) in *Achatina fulica* (mollusca: gastropoda) from Southeast and South regions of Brazil Mem Inst Oswaldo Cruz 2010 105 938 941 21120369 10.1590/s0074-02762010000700019

[B15] Moreira VL Giese EG Melo FT Simões RO Thiengo SC Maldonado A Jr Santos JN Endemic angiostrongyliasis in Brazilian Amazon: natural parasitism of *Angiostrongylus cantonensis* in *Rattus rattus* and *R. norvegicus*, and sympatric giant African land snails, *Achatina fulica* Acta Trop 2012 125 90 97 23072946 10.1016/j.actatropica.2012.10.001

[B16] Simões RO Monteiro FA Sanchez E Thiengo SC Garcia JS Costa-Neto SF Luque JL Maldonado A Endemic angiostrongyliasis in Rio de Janeiro, Brazil Emerg Infect Dis 2011 17 1331 1333 10.3201/eid1707.101822 21762612 PMC3381414

[B17] Ibrahim MM Prevalence and intensity of *Angiostrongylus cantonensis* in freshwater snails in relation to some ecological and biological factors Parasite 2007 14 61 70 10.1051/parasite/2007141061 17432058

[B18] Mahajan RK Almeida AJ Sengupta SR Renapurkar DM Seasonal intensity of *Angiostrongylus cantonensis* in the intermediate host, *Laevicaulis alte* Int J Parasitol 1992 22 669 671 10.1016/0020-7519(92)90017-F 1399252

[B19] Gannon WL Sikes RS Guidelines of the American society of mammalogists for the use of wild mammals in research J Mammal 2011 92 235 253 10.1644/10-MAMM-F-355.1 PMC5909806 29692469

[B20] Lemos ERS D’andrea PS Martins EV, Martins AS, Silva FHAL, Lopes MCM, Moreno MLV, Silva PCT Trabalho com Animais Silvestres Biossegurança, Informação e Conceitos, Textos Básicos 2006 1 Rio de Janeiro: FIOCRUZ 273 288

[B21] Chen HT A new pulmonary nematode of rats, *Pulmonema cantonensis* ng. nsp from Canton Ann Parasitol 1935 13 312 317

[B22] Reiczigel J Rózsa L Quantitative parasitology 3.0 Budapest 2005

[B23] Webster JP Macdonald DW Parasites of wild brown rat (*Rattus norvegicus*) on UH farms Parasitology 1995 11 247 255 7567093 10.1017/s0031182000081804

[B24] Burnham KP Anderson DR Model selection and multimodel inference: a practical information-theoretic approach 2002 New York: Springer

[B25] Thiengo SC Simões RO Fernandez MA Maldonado A Jr *Angiostrongylus cantonensis* and rat lungworm disease in Brazil Hawaii J Med Public Health 2013 72 18 22 23901376 PMC3689498

[B26] Thiengo SC Faraco FA Salgado NC Cowie R Fernandez MA Rapid spread of an invasive snail in South America: the giant African snail, *Achatina fulica* in Brazil Biol Invasions 2007 9 693 702 10.1007/s10530-006-9069-6

[B27] Maldonado A Jr Gentile R Fernandes-Moraes CC D’Andrea PS Lanfredi RM Rey L Helminth communities of *Nectomys squamipes* naturally infected by the exotic trematode schistosoma mansoni in southeastern Brazil J Helminthol 2006 80 369 375 10.1017/JOH2006366 17125546

[B28] Carvalho Odos S Scholte RG Mendonça CL Passos LK Caldeira RL *Angiostrongylus cantonensis* (nematode: metastrongyloidea) in molluscs from harbour areas in Brazil Mem Inst Oswaldo Cruz 2012 107 740 746 10.1590/S0074-02762012000600006 22990962

[B29] Monte TC Simões RO Oliveira AP Novaes CF Thiengo SC Silva AJ Estrela PC Maldonado A Jr Phylogenetic relationship of the Brazilian isolates of the rat lungworm *Angiostrongylus cantonensis* (nematoda: metastrongylidae) employing mitochondrial COI gene sequence data Parasit Vectors 2012 6 248 256 23130987 10.1186/1756-3305-5-248 PMC3514143

[B30] Tokiwa T Harunari T Tanikawa T Komatsu N Koizumi N Tung K-C Suzuki J Kadosaka T Takada N Kumagai T Akao N Ohta N Phylogenetic relationships of rat lungworm, *Angiostrongylus cantonensis*, isolated from different geographical regions revealed widespread multiple lineages Parasitol Int 2012 61 431 436 10.1016/j.parint.2012.02.005 22387862

[B31] Abu-Madi MA Lewis JW Mickail M Et-Nagger ME Behnke JM Monospecific helminth and arthropod infections in an urban area population of brown rats from Doha, Qatar J Helminthol 2001 75 313 320 11818046

[B32] Abu-Madi MA Behnke JM Mikhail M Lewis JW Alkaabi ML Parasite populations in the brown rat *Rattus norvegicus* from Doha, Qatar between years: the effect of host age, sex and density J Helminthol 2005 79 105 111 10.1079/JOH2005274 15946389

[B33] Au ACS Ko RC Changes in worm burden, haematological and serological response in rats after single and multiple *Angiostrongulus cantonensis* infections Z Parasitenkd 1979 58 233 242 10.1007/BF00933930 452645

[B34] Lambert MS Quy RJ Smith RH Cowan DP The effect of habitat management on home‒range size and survival of rural Norway rat populations J Appl Ecol 2008 2008 45 1753 1761

[B35] Zuk M McKean KA Sex differences in parasite infections: patterns and processes Int J Parasitol 1996 26 1009 1023 10.1016/S0020-7519(96)00086-0 8982783

[B36] Schalk G Forbes R Male biases in parasitism of study type, host age and parasite taxon Oikos 1997 78 67 74 10.2307/3545801

[B37] Innes J Advances in New Zealand mammalogy 1990–2000: European rats J R SocNew Zealand 2001 31 111 125 10.1080/03014223.2001.9517642

[B38] Poulin R Sexual inequalities in helminth infections: a cost of being a male? Amer Nat 1996 147 287 295 10.1086/285851

[B39] Schuurs A Verheul HAM Effects of gender and sex steroids on the immune response J Ster Biochem Mol Biol 1990 35 157 172 10.1016/0022-4731(90)90270-3 2407902

[B40] Morales-Montor J Chavarria A De León MA Del Castillo LI Escobedo EG Sánchez EN Vargas JA Hernández-Flores M Romo-González T Larralde C Host gender in parasitic infections of mammals: an evaluation of the female host supremacy paradigm J Parasitol 2004 90 531 546 10.1645/GE-113R3 15270097

[B41] Krasnov BR Morand S Hawlena H Khokhlova IS Shenbrot GI Sex-biased parasitism, seasonality and sexual size dimorphism in desert rodents Oecologia 2005 146 209 217 10.1007/s00442-005-0189-y 16025350

[B42] Lynn DA Brown GR The ontogeny of exploratory behaviour in male and female adolescent rats (*Rattus norvegicus*) Dev Psychol 2009 51 513 520 10.1002/dev.20386 PMC3040845 19582791

[B43] Calhoun JB The Ecology and Sociology of the Norway rat 1963 Bethesda, Maryland: U.S: Department of Health, Education and Welfare

[B44] Taylor KD Range of movement and activity of common rats (*Rattus norvegicus*) on agricultural land J App Ecol 1978 15 663 677 10.2307/2402767

[B45] Brasil. Ministério da Saúde. Secretaria de Vigilância em Saúde. Departamento de Vigilância Epidemiológica Vigilância e Controle de Moluscos de Importância Epidemiológica, Diretrizes Técnicas: Programa de Vigilância e Controle da Esquistossomose 2007 2 Brasília: Editora do Ministério da Saúde

[B46] Reeder DM Kramer KM Stress in free-ranging mammals: integrating physiology, ecology, and natural history J Mammal 2005 86 225 235 10.1644/BHE-003.1

[B47] Himsworth CG Parsons KL Jardine C Patrick DM Rats, Cities, people, and pathogens: a systematic review and narrative synthesis of literature regarding the ecology of rat-associated zoonoses in urban centers Vector Borne Zoonotic Dis 2013 13 1 11 doi:10.1089/vbz.2012.1195 10.1089/vbz.2012.1002 23590323

